# Ezetimibe prevents the development of non-alcoholic fatty liver disease induced by high-fat diet in C57BL/6J mice

**DOI:** 10.3892/mmr.2014.2623

**Published:** 2014-10-10

**Authors:** XIANG WANG, QIAOHUA REN, TAO WU, YONG GUO, YONG LIANG, SUBO LIU

**Affiliations:** 1 Department of Endocrinology and Metabolism, The First Hospital of Shijiazhuang City, Shijiazhuang, Hebei 050011, P.R. China; 2 Department of Urology, The First Hospital of Shijiazhuang City, Shijiazhuang, Hebei 050011, P.R. China; 3 Department of Preventive Medicine, The First Hospital of Shijiazhuang City, Shijiazhuang, Hebei 050011, P.R. China

**Keywords:** non-alcoholic fatty liver disease, hepatocellular carcinoma, liver steatosis, insulin resistance, ezetimibe

## Abstract

There is currently no established treatment for non-alcoholic fatty liver disease (NAFLD), including its most extreme form, non-alcoholic steatohepatitis (NASH). Ezetimibe, an inhibitor of Niemann-Pick C1 Like 1-dependent cholesterol absorption, improves diet-induced hyperlipidemia and attenuates liver steatosis and insulin resistance. The aim of the present study was to determine whether ezetimibe treatment is able to inhibit the development of NAFLD, and to elucidate the underlying mechanism, using C57BL/6J (B6) mice maintained on a high-fat diet. Male B6 mice (20 weeks of age) were divided into the following two groups (n=7 in each group): Mice fed a high-fat diet for four weeks and mice fed a high-fat diet with 0.0064% (wt/wt) ezetimibe (5 mg/kg/day) for four weeks. Administration of ezetimibe significantly reduced liver steatosis and fibrosis. Ezetimibe reduced serum cholesterol, hepatic fat accumulation and insulin resistance in the liver of mice fed the high-fat diet. Furthermore, ezetimibe significantly reduced hepatic mRNA expression of *Acc1* and *Scd1*, which are involved in hepatic fatty acid synthesis. Ezetimibe significantly reduced hepatic *Cd36* gene expression, upregulation of which is significantly associated with insulin resistance, hyperinsulinemia and increased steatosis. The protein expression of SKP2, a viable therapeutic target in human cancer, was also reduced by ezetimibe. These findings suggest that ezetimibe may be an effective therapy for high fat-induced NAFLD, including NASH.

## Introduction

Non-alcoholic fatty liver disease (NAFLD) is a clinicopathological syndrome in which the severity can range from simple fatty liver to non-alcoholic steatohepatitis (NASH), cirrhosis and hepatocellular carcinoma, and is observed in patients with no history of excessive alcohol consumption (1). A previous study has indicated that NAFLD can coexist with type 2 diabetes mellitus, obesity and dyslipidemia. In particular, hyperlipidemia and insulin resistance were implicated in the initiation and progression of NAFLD (2). Currently, an increase in energy consumption by exercise and/or a reduction in energy intake are accepted methods for the prevention of NAFLD. No standard treatments are currently used to reverse NAFLD, and effective medical interventions have focused on diet control and exercise. There remains a requirement for the development of effective pharmacological agents, due to the increasing prevalence of NAFLD.

Ezetimibe remains the most widely used first-line drug for the treatment of hypercholesteremia. Ezetimibe exerts its effect predominantly by inhibiting cholesterol absorption and it was demonstrated to block Niemann-Pick C1 Like 1-mediated cholesterol absorption at the brush border of the intestine and the liver (3). In addition to improving hypercholesterolemia in patients with dyslipidemia, attention has recently been drawn to its potential attenuation of liver steatosis (4,5). It has been shown that Ezetimibe may exert these effects by reducing *Srebp-1c* expression in mice fed a high-fat diet (6). Another study demonstrated that ezetimibe was effective for reducing serum low-density lipoprotein cholesterol levels resistant to lifestyle intervention in patients with non-alcoholic fatty liver disease (7). Hepatic steatosis, induced by a high-fat diet, but not a high-fructose diet, was inhibited by ezetimibe administration (8). It has also been reported that hepatic iron levels in mice fed a high-fat diet are increased following treatment with ezetimibe (9). Despite these notable findings, mechanisms by which ezetimibe administration ameliorates hepatic steatosis, insulin resistance and obesity remain largely unexplored.

NAFLD is considered as the hepatic manifestation of the metabolic syndrome that is closely associated with obesity, hyperlipidemia, type 2 diabetes mellitus and insulin resistance. The initiation and progression of metabolic syndrome (MS) is mainly associated with the consumption of high-fat diets and/or high-carbohydrate diets. Epidemiological studies suggest that consumption of high-fat diets (≥30% of energy from fat) is associated with a high prevalence of being overweight, central obesity and MS (10,11). Rodents fed a high-fat diet closely mimic a number of the features observed in humans with NAFLD, and present with obesity, impaired glucose tolerance, dyslipidemia and fat accumulation in the liver (12,13). In the current study, the effects of ezetimibe on an HF-induced mouse model C57BL/6J (B6) for NAFLD were investigated.

## Materials and methods

### Animals

Male B6 mice (14 weeks old) were purchased from the Hebei Medical University, Center for Animal Experimentation (Shijiazhuang, China) and were maintained in the animal facilities of Hebei Medical University with standard animal care procedures based on the institutional guidelines. The mice were fed a normal laboratory diet (22.3% protein, 6.2% fat, 3.0% fiber, 6.5% ash and 47.8% complex carbohydrate) with free access to water, and were housed with a regular 12-h light/dark cycle according to the Hebei Medical University Guidelines for the Care and Use of Laboratory Animals. Following acclimatization for two weeks, B6 mice were fed a high-fat chow (High-Fat Diet 32; CLEA Japan, Inc., Tokyo, Japan) for four weeks, then the mice were divided into two groups (n=7/group); those fed the high-fat chow for four weeks (HF group), and those fed the high-fat chow with 0.0064% wt/wt ezetimibe (5 mg/kg/day) for four weeks (HF+EZ group). After 16-h fasting, all mice were sacrificed under anaesthesia by intraperitoneal administration of pentobarbital (60 mg/kg body weight; Nembutal; Dainippon Sumimoto Pharma Co., Ltd., Osaka Japan) and medetomidine (0.3 mg/kg body weight; Domitor; Meiji Seika Kaisha, Ltd., Tokyo, Japan). The ezetimibe was provided by Merck Sharp & Dohme (Whitehouse Station, NJ, USA).

### Phenotype determination

Alanine aminotransferase (ALT), total serum cholesterol and triglyceride (TG) were measured as described previously (14).

### Measurement of liver triglyceride

Liver triglyceride content was analyzed using a Triglyceride Quantification Kit (#ab65336; Abcam, Cambridge, MA, USA) as previously described (14).

### Intraperitoneal glucose tolerance test (ipGTT)

The mice were given an ipGTT (2 g glucose/kg body weight) subsequent to overnight fasting. The glucose levels were measured after fasting prior to glucose administration (0 min), and at 30, 60, 90 and 120 min post-glucose load.

### Histological examination of liver

To study histological changes, liver tissue samples were formalin-fixed and paraffin-embedded (Santa Cruz Biotechnology, Inc., Dallas, TX, USA) and subjected to hematoxylin-eosin (H&E; Biocare Medical, LLC., Concord, CA, USA) and Sirius red (Direct Red 80; Sigma-Aldrich, St. Louis, MO, USA) staining. All the images were acquired and analyzed using a BZ-8000 Fluorescence Microscope (Keyence Corporation, Osaka, Japan). H&E-stained sections and Sirius red-stained sections were graded according to the NASH activity score and the fibrosis score as previously described (15,16). The evaluation was performed by two experienced pathologists who were blinded to the treatments that the mice had received, according to methods previously described (16).

### Hepatic gene expression analysis

Total RNA was extracted from frozen liver samples using Isogen (Nippon Gene Co., Ltd., Tokyo, Japan), and cDNA was synthesized using a High Capacity cDNA Reverse Transcription kit (Applied Biosystems Life Technologies, Carlsbad, CA, USA). Quantitative polymerase chain reaction (qPCR) was performed using TaqMan^®^ Gene Expression Master Mix (Applied Biosystems Life Technologies) with a previously described method (17). The expression of the following genes was evaluated with probes from Applied Biosystems Life Technologies as follows: *Acc1* (Mm01304282_m1) for acetyl-coenzyme A (CoA) carboxylase 1; *Srebf1* (Mm00550338_m1) for sterol regulatory element binding protein (SREBP)-1c; *Scd1* (Mm00772290) for stearoyl-CoA desaturase 1; *Fasn* (Mm00662319_m1) for fatty acid synthase; *Srebf2* (Mm01306289_m1) for SREBP-2; *Cd36* (Mm00432403) for the fatty acid translocase (FAT); *Dgat2* (Mm00499530) for diacylglycerol O-acetyltransferase 2; *Bax* (Mm00432448_m1) for Bcl-2 associated X protein; *Bcl-2* (Mm00477631_m1) for B cell lymphoma-2; *Cpt1a* (Mm00550438_m1) for carnitine palmitoyltransferase 1A; *Ppara* (Mm00440939_m1) for peroxisome proliferator-activated receptor α; *ApoB* (Mm01545164_m1) for apolipoprotein B; *Mttp* (Mm00435015_m1) for microsomal triglyceride transfer protein (MTP); *Ccl2* (Mm00441242_m1) for chemokine (C-C motif) ligand 2; *Emr1* (Mm00802530_m1) for cell surface glycoprotein F4/80; *Tnf* (Mm00443258_m1) for tumor necrosis factor-α; and *Tgfb1* (Mm00441724_m1) for transforming growth factor β-1. All experiments were performed in duplicate and all gene expression levels were normalized to *Hprt1* expression (Mm00446968_m1).

### Western blot analysis

Liver samples were collected and proteins were separated by SDS-PAGE (10% Mini-Protean^®^ TGX™ gel and Mini-Protean^®^ Tetra Cell Mini Trans-Blot module; Bio-Rad Laboratories, Hercules, CA, USA), and blotted onto polyvinylidine fluoride membranes (Bio-Rad Laboratories). The blots were incubated with polyclonal anti-rabbit SKP2 (L70; #4313; 1:1,000) or β-actin primary antibodies (#4967, 1:1,000; both from Cell Signaling Technology, Inc., Danvers, MA, USA) overnight at 4°C using slow rocking and the horseradish peroxidase (HRP)-conjugated secondary antibody, anti-rabbit IgG-HRP (1:1,500; Cell Signaling Technology, Inc.). Thereafter, the membranes were visualized by enhanced chemiluminescence, and the signals were quantified as previously described (16).

### Statistical analysis

All data are expressed as the means ± standard deviation. Statistical comparisons were made using the two independent-samples t-test and Mann-Whitney U test. P<0.05 was considered to indicate a statistically significant difference. All statistical analyses were performed using SPSS for Windows, version 18 (SPSS, Inc., Chicago, IL, USA).

## Results

### Physiological characteristics

**Figure 1:**
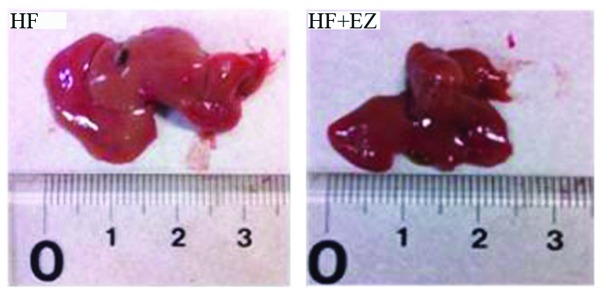
Macroscopic findings observed following short-term treatment with ezetimibe (EZ) in C57BL/6J mice fed a high-fat chow (HF).

**Figure 2:**
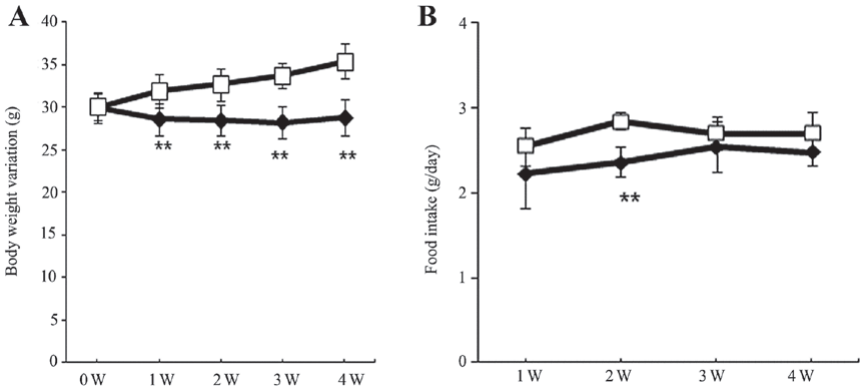
Food consumption and body weight variation of C57BL/6J mice fed high-fat chow. Variations in (A) body weight and (B) food consumption. White squares, mice fed high-fat chow; black circles, mice fed high-fat chow plus ezetimibe. The data are expressed as the means ± standard deviation (n=7). ^**^P<0.01, vs. HF group. W, week.

The livers of HF mice were markedly enlarged and exhibited a paler color as compared with the livers of the HF+EZ group (Fig. 1, Table I) The food consumption and body weight of the two groups were monitored throughout the observation period. Although the baseline body weight was similar between the two groups (Table I, Fig 2A), the body weight was significantly lower in the HF+EZ group as compared with the HF group during weeks 1–4 following ezetimibe treatment (P<0.01; Fig. 2A). Food consumption was similar between HF and HF+EZ groups (Fig. 2B). The final body and liver weights were significantly lower in the HF+EZ group than in the HF group (P<0.01; Table I). Liver TG content was significantly lower in the HF+EZ group, as compared with that of the HF group (P<0.05; Table I).

### Histological changes in the liver

**Figure 3:**
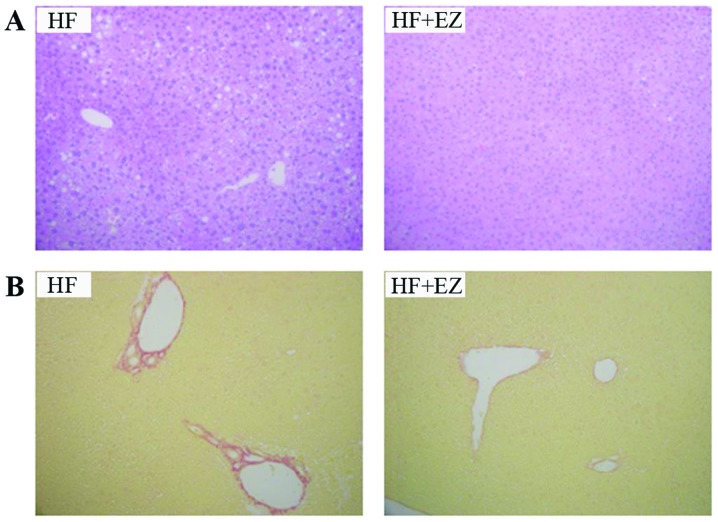
Histological appearance of the liver from C57BL/6J mice fed high-fat chow (HF). Liver sections from mice fed HF and HF plus ezetimibe (EZ). (A) Hematoxylin and eosin staining indicating reduced lipid deposits and (B) Sirius red staining indicating reduced fibrosis in the HF+EZ group, as compared with the HF group. Magnification, ×200.

Lipid deposits in the liver were smaller in the HF+EZ group as compared with those in the HF group (Fig. 3A). Regarding the NASH activity score, the HF group had a total score of 2.3±0.7, while the HF+EZ group had a total score of 1.0±0.5, indicating a significant difference between the two groups (P<0.01; Table I). Sirius red staining of the liver revealed that the HF+EZ group exhibited a lower degree of liver fibrosis as compared with the HF group (Fig. 3B). The fibrosis score was significantly different between the two groups (1.0±0.2 in the HF group vs. 0.6±0.1 in the HF+EZ group; P<0.01; Table I).

### Glucose tolerance

**Figure 4:**
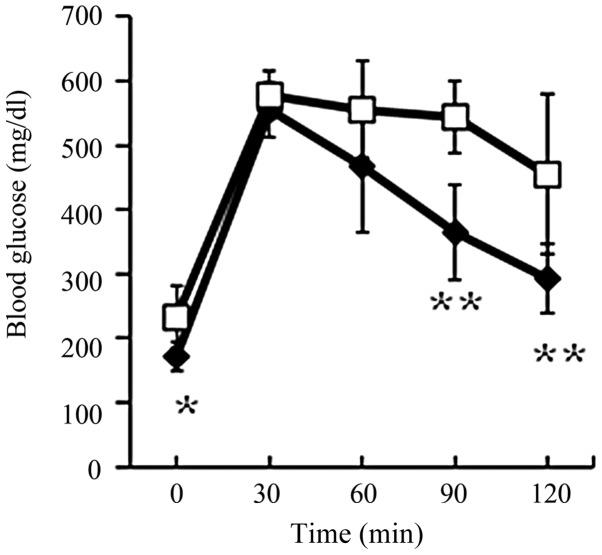
Effect of ezetimibe (EZ) on glucose metabolism. Glucose levels during an intraperitoneal glucose tolerance test were analyzed in C57BL/6J mice fed high-fat chow. White squares, mice fed HF; black rhombuses, mice fed HF plus ezetimibe. The data are expressed as the mean ± standard deviation (n=7). ^*^P<0.05 and ^**^P<0.01, vs. HF group.

The fasting glucose level in the HF+EZ group was significantly lower than that of the HF group (P<0.05; Fig. 4). In addition, the blood glucose level at 90 and 120 min during the ipGTT were significantly lower in the HF+EZ group as compared with the HF group (P<0.01; Fig. 4).

### Serum biochemical markers

Serum total cholesterol levels in the HF+EZ group were significantly lower than that of the HF group (P<0.05; Table I). Serum ALT and TG levels in the HF+EZ group were not significantly lower than those in the HF group (Table I).

### Hepatic gene expression for lipid metabolism, inflammation, fibrosis and apoptosis

**Figure 5:**
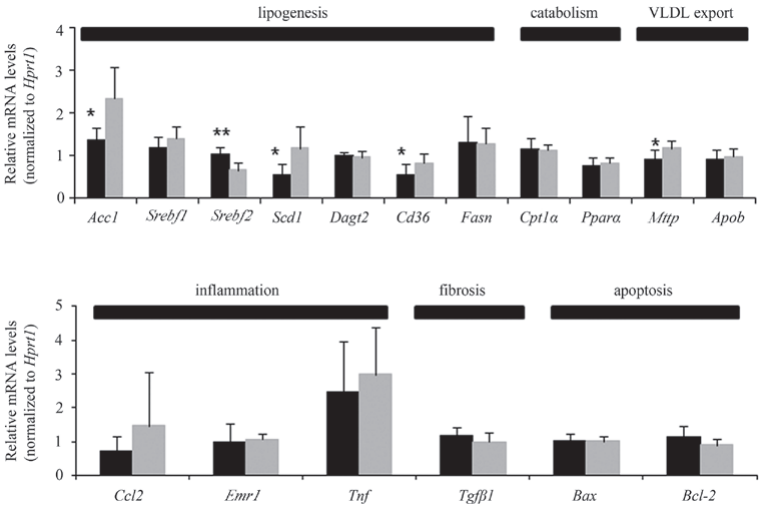
Effect of ezetimibe (EZ) treatment on hepatic gene expression. Gene expression analysis by quantitative polymerase chain reaction from the livers of mice fed high-fat chow (HF) plus EZ treatment (black bars) and HF group (gray bars). The mRNA levels are expressed relative to those in the HF group, as the mean ± standard deviation, following normalization against the expression of *Hprt1* mRNA (n=7). ^*^P<0.05 and ^**^P<0.01, vs. HF group.

The mRNA levels of specific hepatic lipogenesis-related genes were evaluated, including the following: *Acc1*, *Srebf1*, *Srebf2*, *Scd1*, *Dagt2*, *Cd36* and *Fasn*. Hepatic expression levels of *Acc1*, *Scd1* and *Cd36* were significantly lower in the HF+EZ group, compared with the HF group (P<0.05; Fig. 5), while those of *Srebf2* were significantly higher (P<0.01). Notably, the hepatic expression level of *Mttp* was also significantly lower in the HF+EZ group, as compared with the HF group (P<0.05). Expression levels of genes associated with lipid catabolism, fibrosis and apoptosis were not significantly different between the two groups. Regarding the expression of genes involved in inflammation, *Ccl2* and *Tnf* were slightly lower in the HF+EZ group than in the HF group, however this was not significant, and there was no difference in the expression of *Emr1* between the two groups (Fig. 5).

### Western blot analysis of SKP2

**Figure 6:**
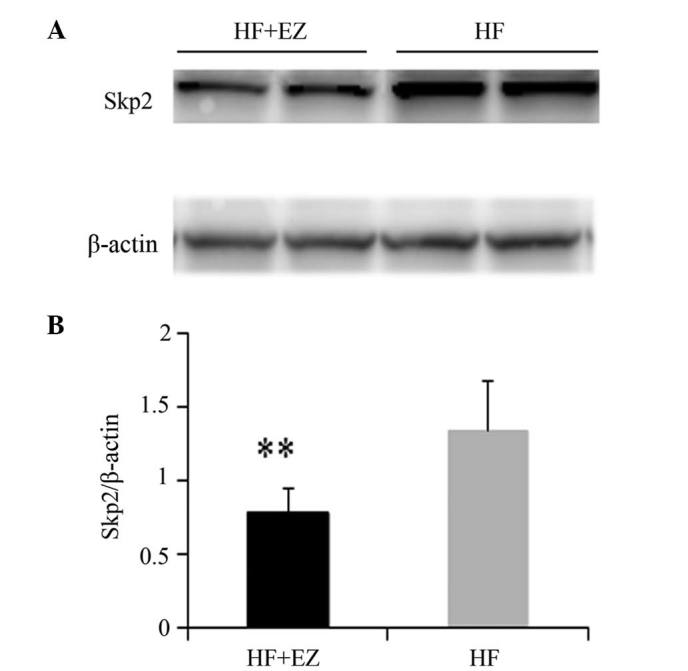
Effect of ezetimibe (EZ) treatment on hepatic protein expression. (A) Western blot analysis of SKP2 in livers from the high fat chow (HF) and HF+EZ groups, and (B) densitometric quantification of SKP2/β-actin expression in livers from the HF+EZ (black bars) and HF (gray bars) groups. The data represent the means ± standard deviation (n=7). ^**^P<0.01, vs. HF group.

The protein expression level of SKP2 in the HF+EZ group was significantly lower than that in the HF group (P<0.01; Fig. 6).

## Discussion

NAFLD is a growing public health concern in developed and developing countries due to the increasing prevalence of obesity, diabetes and the metabolic syndrome induced by the excessive consumption of high-fat and cholesterol-containing diets and the lack of exercise in the general population.

In the present study, the effects of ezetimibe on NAFLD were investigated using a high-fat induced mouse model. The results demonstrated that ezetimibe significantly reduced the NASH activity and fibrosis score in mice fed the high-fat diet compared with the untreated mice, indicating favorable effects of ezetimibe on liver steatosis and fibrosis. In addition, ezetimibe improved serum cholesterol, hepatic fat accumulation and insulin resistance in the livers of mice fed a high-fat diet. Furthermore, ezetimibe significantly reduced mRNA levels of *Acc1*, *Scd1* and *Cd36* in the liver, which are all associated with hepatic lipogenesis and free fatty acid transportation. The level of SKP2 protein upregulation, which induces uncontrolled cell proliferation and tumor progression, was also reduced by ezetimibe.

Ezetimibe administration reduced the body and liver weights. The current study considered the possibility that the effect of ezetimibe may be mediated by food intake, as reduced food intake would significantly affect body weight, and therefore influence hepatic steatosis. However, the reduced body and liver weights were not associated with reduced food consumption in the present study, and weight-specific food intake was indicated to be similar between the two groups. This suggests that ezetimibe directly protected against obesity and hepatic steatosis, independent of food intake (18).

A previous study in C57BL/6 mice demonstrated that fat overconsumption is key in the etiology of hepatic steatosis (19). Another study suggested that lipid accumulation in the liver of mice and rats can be induced by a high-fat diet (20). Long-term fat overconsumption may increase the risk of insulin resistance and obesity, which enhance susceptibility to NAFLD. In rodent models and humans, hepatic steatosis is consistently associated with the development of hepatic insulin resistance (2). In the current study, ezetimibe ameliorated insulin resistance in mice fed the high-fat diet, and significantly altered fasting glucose in addition to glucose levels at 90 and 120 min of the ipGTT, which strongly indicated that ezetimibe improved insulin sensitivity. Ezetimibe administration may inhibit high fat-induced insulin resistance by reducing intestinal fat absorption and weight gain, rather than via downregulation of *Srebf1* as suggested previously (6,21).

CD36 functions in the development of hepatic steatosis in rodents, and is the most well-characterized free fatty acid transporter. Thus far, the significance of CD36 in human liver diseases remains unclear. Hepatic expression of CD36 is abnormally increased in non-alcoholic fatty liver disease, and a previous study indicated that hepatic CD36 upregulation was significantly associated with insulin resistance, hyperinsulinemia and increased steatosis in patients with NAFLD (22). Hepatic CD36 expression is normally low, but its expression is increased in rodents with fatty liver (23). Additionally, another study demonstrated that CD36 mRNA levels increased concomitantly with hepatic TG content in a number of animal models of fatty liver (24,25). Notably, experimental amelioration of steatosis in mice was accompanied by hepatic CD36 downregulation (26,27). Modulation of CD36 expression in hepatocytes may prove useful for the prevention or treatment of liver fat accumulation in patients with NAFLD. In the present study, ezetimibe significantly reduced CD36 gene expression in the liver. Hence, ezetimibe may ameliorate hepatic insulin resistance in addition to dyslipidemia and hepatic steatosis, partly via a pathway involving CD36 in high-fat diet-induced B6 mouse models of NAFLD.

SREBP-1c is a key transcriptional activator of lipogenesis, and is responsible for regulating genes involved in lipogenesis, including *Acc1*, *Fas* and *Scd1* (5). In the present study, ezetimibe treatment reduced the mRNA expression levels of *Acc1* and *Scd1*, which were correlated with hepatic lipogenesis, and upregulated the gene expression of *Srebf2*, as previously reported (6). Inhibition of cholesterol absorption may occur by ezetimibe-activated hepatic expression of SREBP-2, which is established as a key regulator of cholesterol synthesis and uptake (28). The hepatic mRNA expression level of *Mttp* was increased in the HF group compared with the level in the HF+EZ group in the present study, suggesting a compensatory mechanism to release excess lipid as VLDL, as previously suggested (29).

Overexpression of SKP2, a positive regulator of G1-to-S phase transition, has been observed in numerous cases of human cancer, including hepatocellular carcinoma (HCC) (30,31). Previous reports have demonstrated in various types of cancer, that SKP2 mRNA or protein expression is increased compared with normal tissues (32–35). Uncontrolled SKP2 upregulation may favor cell transformation *in vitro* and tumor progression (36,37). It has been reported that a mouse knockout for *Skp2* results in a reduction of cell proliferation and mouse body size (38). Another study indicated that silencing of *Skp2* by siRNA in HuH7 and HepG2 cells led to growth restraint, enhanced apoptosis, and a rise in protein levels of cell cycle inhibitors, with consequent reduction of their ubiquitination (30). Other studies have indicated that *Skp2* serves as an oncogene in HCC and thus is upregulated by increased transcriptional activity (39,40). Therefore, the *Skp2* gene may be a therapeutic target for NAFLD-related HCC. In the present study, the protein expression of SKP2 was reduced by ezetimibe administration, providing a clue that ezetimibe may prevent the progression of NAFLD-related HCC through downregulation of SKP2 protein expression. However, further research is required to confirm this contention.

In conclusion, ezetimibe administration in an HF-induced model of NAFLD resulted in lower serum cholesterol levels, and amelioration of glucose tolerance, histological lesions and hepatic expression of lipogenesis-related genes. In addition, B6 mice that received ezetimibe treatment presented lower *Cd36* gene expression in the liver, suggesting ezetimibe may ameliorate hepatic insulin resistance in addition to dyslipidemia and hepatic steatosis, in part via a pathway involving *Cd36*. Furthermore, the protein level of SKP2, a therapeutic target for NAFLD-related HCC, was reduced by ezetimibe administration. These data suggest that ezetimibe may have the potential to be used as an effective drug for NAFLD and NAFLD-related HCC.

## Figures and Tables

**Table I:** Characteristics of C57BL/6J mice fed a high-fat diet with or without ezetimibe.

	C57BL/6J	
	
Variable	HF	HF+EZ
Starting body weight (g)	30.0±1.6	30.0±1.8
Final body weight (g)	35.4±2.0	28.8±2.1^b^
Liver weight (mg)	1026±63	858±47^b^
Liver weight/body weight	0.030±0.001	0.032±0.003
NASH activity score	2.3±0.7	1.0±0.5^b^
Fibrosis score	1.0±0.2	0.6±0.1^b^
Serum ALT (IU/L)	35.7±8.5	30.9±7.2
Serum total cholesterol (mg/dl)	119.6±11.3	90.0±28.5^a^
Serum triglyceride (mg/dl)	14.7±4.6	12.0±3.7
Liver TG content (mg/g liver)	16.2±1.4	13.5±1.5^a^

The data are expressed as the means ± standard deviation; n=7.

a P<0.05 and

b P<0.01, HF vs. HF+EZ.

HF, high-fat chow; EZ, ezetimibe; NASH, non-alcoholic steatohepatitis; ALT, alanine aminotransferase; TG, triglyceride.

## Abbreviations

MTP: microsomal triglyceride transfer protein
NASH: non-alcoholic steatohepatitis
NAFLD: non-alcoholic fatty liver disease
HCC: hepatocellular carcinoma
EZ: ezetimibe
HF: high fat diet
TG: triglyceride
Chol: cholesterol
B6 mouse: C57BL/6J mouse
ALT: alanine aminotransferase
